# Pervasive Decision Support Systems in Healthcare Using Intelligent Robots in Social Media

**Published:** 2017-01

**Authors:** Taha SAMAD-SOLTANI, Peyman REZAEI-HACHESU, Marjan GHAZISAEEDI

**Affiliations:** 1. Dept. of Health Information Technology and Management, School of Allied Medical Sciences, Tehran University of Medical Sciences, Tehran, Iran; 2. Dept. of Health Information Technology, School of Health Management and Informatics, Tabriz University of Medical Sciences, Tabriz, Iran

## Dear Editor-in-Chief

In social world, the healthcare provider must become culturally competent with various and diverse populations. Now in the growing technology age, there is even more expanding diversity within the online social media and communities. With the advancement and daily uses of technology, there is a growing interest in researching what other areas technology can impact. Social media provide a strong medium to be applied by the society, patients, and health providers to interact about health problems with the possibility of potentially promoting health outcomes and this is a new dimension in healthcare. Social media is a pervasive tool, which makes collaboration between users (1).

Telecommunications may provide some strategies in addressing barriers, such as access to care and cost. It is important to understand that telehealth services can be expanded to fit the requirements of the users, such as sharing various medium, online chat or store and forward messages, voice call, or the growing usage of smart-phone devices to reduce costs and travel distance by providing a link to more convenient access to services (2). ‘Digital health’ is a pervasive implication that lacks scientific definition and terminology. This wide field includes Mobile Health, Wireless Health, Health 2.0, eHealth, e-Patient, Healthcare Health IT, Big Data, Health Data, Cloud Computing, Quantified Self, Wearable Computing, Gamification, and Telehealth/Telemedicine. Social media provide the ability of the creation and sharing of user content. Social media is an umbrella term for a broad range of technologies including blogs and micro-blogs (WordPress, Google Blog, Twitter); social networking sites (Facebook, Pinterest, LinkedIn); collaborative projects (Wikipedia); content communities (YouTube, Pinterest, Instagram); virtual social worlds (Second Life), virtual gaming worlds (World of Warcraft) and Social Instant Messaging apps (Telegram, Viber). By using these medium, patients can generate and share content related to health education and research, information and networking and tracking personal progress (3). The growth of telehealth parallels the development of technology from closed circuit television, personal computers and broadband Internet to smartphones in the 21st century. Advanced hardware coupled with innovative software significantly impacts health delivery. At the social level, telehealth impact healthcare utilization and improve access to care. At the individual level, telehealth usecases such as monitoring, education and self-care training can promote health outcomes (4).

Telegram is an open source app, used by more than 100 million people worldwide and 23 million in Iran (2016) that allows smartphone users to send text messages and other types of media (such as videos, voice messages, and photographs) to their contacts. It also facilitates the creation of groups and channels; this allows multiple users to participate in and monitor the conversation. Telegram avoids charging for each message by using cellular data plans and wireless Internet networks and is fully free. It is very secure and reliable platform. It is cloud based and has a new intelligent approach to developers in designing intelligent agents over this cloud. The name of this new technology is telegrambots or telegram robots. Robots and agents differ from ordinary computer programs in that they have autonomy, interact with the environment, and initiate tasks. The marriage of artificial intelligence and computer science has made possible robots and agents with logical features like humans, such as gestures and speech. ‘Agent’ refers to a software module (5). Telegram robots or bots are special accounts that do not require an additional phone number to set up. These accounts serve as an automatic interface for code running somewhere on your server. In the behind of scenes, you can program any code with some integrated development kits and any supported libraries and modules such as artificial intelligence.

Despite of regular apps for smartphones in various platforms, which needs to install separately according their domain and user interests, Social instant messaging apps, especially telegram, install once and then provides numerous services on cloud. They can contribute in various tasks such as teach, play, search, broadcast, remind, connect, and integrate with other services, or even pass commands to the Internet of Things. This valuable advantage lead to pervasive services with no more trouble to users and complexity of services hide in server-side. In apps like telegram, incremental process in evolution of services and capabilities automatically was distributed on user smartphone. Keep in mind that if a DSS is designed to diabetes consultation in the form of an intelligent bot, all users which want to get information or decision support about diabetes, can access to this service without doing extra work and only by adding bots to their contacts and interact with him. These robots applied MTProto encryption protocol to safe and confident messaging between users and bots and we can satisfy security essentials emphasized in DSS development.
